# A contrast of meta and metafor packages for meta‐analyses in R

**DOI:** 10.1002/ece3.6747

**Published:** 2020-09-14

**Authors:** Christopher J. Lortie, Alex Filazzola

**Affiliations:** ^1^ Department of Biology York University Toronto ON Canada; ^2^ The National Center for Ecological Analysis and Synthesis UCSB Santa Barbara CA USA; ^3^ Biological Sciences University of Alberta Edmonton AB Canada

**Keywords:** computational tools, contrasts, meta, meta‐analyses, metafor, methods, R, review

## Abstract

1. There is extensive choice in R to support meta‐analyses.
2. Two packages in this ecosystem include meta and metafor and provide an excellent opportunity to apply a structured checklist previously developed for contrasts between R packages relevant to challenges in ecology and evolution.
3. Meta is a direct, intuitive choice for rapid implementation of general meta‐analytical statistics. Metafor is a comprehensive package best suited for relatively more complex models.
4. Both packages provide estimates of heterogeneity, excellent visualization tools, and functions to explore publication bias.
5. The package metafor has a steeper learning curve but greater rewards. Reference to the learning curve and capacities of the statistical software Stata provided a benchmark outside the R ecosystem and confirmed the consistency in statistics.
6. The usefulness of meta‐analyses is not just in the synthesis of the research but in the process of doing the scientific synthesis. Reporting of contrasts and checks for robust statistics is an important contribution to more transparent and reproducible scientific syntheses.

## Graphical abstract

Meta‐analyses are common and increasing in ecology and evolution. Here, we contrast two common R packages used in our field and further provide an explanation and contrast with another common tool.

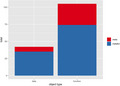

## 1 INTRODUCTION

Meta‐analyses are common and powerful synthesis tools in science. Typically in the natural sciences, meta‐analyses are used as a mechanism to describe and aggregate quantitative evidence from a set of peer‐reviewed, primary research publications (Nakagawa, Noble, Senior, & Lagisz, 2017). The term meta‐analysis in the natural sciences is used to describe synthesis studies that comprise an analysis of effect sizes with statistics examining intervention efficacy (Vetter, Rücker, & Storch, 2013). In other fields, the terms systematic review and meta‐analysis are used more interchangeably, and meta‐statistics are often done on compiled randomized controlled trials or other relatively large datasets in addition to data derived from peer‐reviewed publications. Effect sizes are primarily used in ecology and evolution in meta‐analyses from the mean values and variance estimates from publications (Stewart & Schmid, 2015), but this is changing because of computation (Carey et al., 2019) and thresholds crossed in open big data (Hampton et al., 2013; Kenall, Harold, & Foote, 2014). The statistics were commonly done using MetaWin (Rosenberg, Adams, & Gurevitch, 2000) or other GUI‐based applications for a number of years in ecology and evolution. More recently, however, statistics in the fields of ecology and evolution for instance have increasingly moved to the programming language R (Lai, Lortie, Muenchen, Yang, & Ma, 2019). Synthesis statistics are no exception. At least two R packages have risen to prominence for general meta‐analytical statistics in the sciences—namely meta (Schwarzer, 2007, 2019) and metafor (Viechtbauer, 2010, 2017). The earliest published and indexed descriptions of each package have been cited 757 times (meta, Scopus) and 4,390 times (metafor, Web of Science), respectively. Download statistics for the packages from the Comprehensive R Archive Network (CRAN) also confirms their relatively high‐frequency use with 279,975 meta downloads and 396,067 metafor downloads (Lortie Christopher & Filazzola, 2020). Given that meta‐analyses are also increasingly published in these same fields (Cadotte, Mehrkens, & Menge, 2012; Lortie & Bonte, 2016), a brief comment on the ecosystem of analytical choices that R provides is beneficial and timely. We need synthesis to inform evidence‐based decisioning, and meta‐analyses can be a fundamental tool if aggregated primary datasets are unavailable. Furthermore, even with primary data in hand, data reduction to effect sizes within primary and synthesis studies is a mechanism to illustrate differences and strength of effects. These approaches provide the capacity for higher‐order analyses and reuse (Gerstner et al., 2017) suggesting that familiarity with effect sizes is both germane and practical.

## 2 THE R ECOSYSTEM FOR META‐ANALYSES

Like many fundamental challenges in science, the R developer community provides potential solution sets distributed across multiple packages for synthesis. Broadly speaking, alternative packages in R sometimes examine an issue from different perspectives and provide unique functions. In other instances, packages can be very similar or analogs in terms of functionality and use conceptually aligned functions that differ only in nomenclature or arguments. Here, we apply a checklist recently developed to facilitate and structure these contrasts (Lortie, Braun, Filazzola, & Miguel, 2020). Scientific synthesists that choose to do a meta‐analyses in R have options. A total of 63 packages associated with various aspects of conducting a meta‐analysis have been identified in a comprehensive review and typology of options (Polanin, Hennessy, & Tanner‐Smith, 2016). Both meta and metafor are among 11 generic packages identified in this comprehensive review. These two packages are analogs but with different inherent workflows. The package metagear is also a powerful R package that can augment many aspects of meta‐analysis including review and retrieval of literature, effect size calculations, and replication analyses (Lajeunesse, 2016). There is also rmeta for simple fixed and random effects meta‐analyses (Lumley, 2018), mada for diagnostics (Doebler, 2017), netmeta for frequentist network meta‐statistics (Doebler, 2017), and mvmeta for multivariate‐derived data aggregations (Gasparrini, 2018) to name a few options relevant to the ecology and evolution community. The latter three packages listed have distinct and specific niches for analysis while meta and metafor overlap considerably. Consequently, a brief contrast is provided here for three reasons. Firstly, this is an application of the checklist developed for contrasting R packages (Lortie et al., 2020), and we hope that it is a worthwhile example to consider adding to the analyses and reporting workflows in our field (Table 1). Secondly, the purpose is not to be prescriptive but instead offer a novel framework to consider when about to engage in a synthesis such as a meta‐analysis. This is particularly relevant to the ecology and evolution community because field and laboratory research is not always viable, and synthesis is a valid research activity that can also be improved through transparency by explaining how you choose your tool and maybe even test different ones including those outside R. Thirdly, this short piece is intended to describe a few ways to approach the tools for meta‐analyses and stimulate further discourse in an already positively engaged community of practitioners.

**TABLE 1:** A contrast of meta and metafor using the critical principles developed in ‘A checklist for choosing between R packages in ecology and evolution’ (Lortie et al., 2020)

Item	Criteria	Meta	Metafor	Stata
1	Maturity	Fourth major version	Third major version	Version 16
2	Active development	Schwarzer maintains website and GitHub repository	Viechtbauer maintains website and GitHub repository	Blog, numerous releases and updates, and extensive development team
3	Recently updated	20 February 2020	19 March 2020	December 2019
4	Documentation available	Reference manual	Reference manual and two vignettes	Reference manual, peer‐reviewed publications, and a Stata Journal published quarterly
5	Published in similar projects	Widespread use in ecology, evolution, and to a lesser extent the social sciences	Widespread use in ecology, evolution, and the social sciences	Common in many disciplines including the social sciences
6	License	GPL‐2	GPL‐2	Single user to network licensing, fee based
7	Semantics intuitive	Functions to fit meta‐analyses corresponds to data type intuitive	Fitting meta‐analyses logic is similar to GLM fitting in R	Workflow described in manual aligns with best practices in meta‐analysis literature
8	Functions that get the job done	Most major data types have a specific function and there is also a generalized metagen function	Requires clear specification in arguments but rma function is a very general yet flexible tool	Reasonable collection of features and functions to fit different meta‐analyses, tools to explore bias and heterogeneity
9	Arguments to support your needs	There is an sm argument for specification of summary measures within meta‐analysis functions	Extensive breadth in capacity to specify model fitting	Standard set of tools to explore synthesis data
10	Dependencies reasonable and reported	Depends on R, and imports include grid, metafor, lme4, and CompQuadForm listed	Depends on R, and imports include stats, utils, graphics, grDevices, and nlme	Major desktop operating systems supported but requires relatively recent OS versions

## 3 CONTRASTS

Meta is a well‐maintained, recently updated CRAN R package (Version 4.11‐0 updated on Feb 20, 2020) with 33 unique functions, 7 sample datasets (Appendix S1), and a reference manual. There is also a thorough textbook devoted to meta‐analysis in R that focuses primarily on this package with descriptions of use, theory, and examples provided (Schwarzer, Carpenter, & Rücker, 2015). It is highly capable of resolving most general meta‐analytical challenges that an analyst will face including the capacity to include Empirical Bayes estimators as arguments in some functions, predictive meta‐statistics, interaction terms, meta‐regression, and modifiers. The package metafor is a dependency for many of the functions in meta including the rma.uni, rma.glmm, or rma.mv functions that are sourced internally from calls by the meta package. This is important to the user that will review the functions or the underlying maths but does not impact the user if she prefers the structure of the arguments within functions, the semantics, or the workflow of the meta package. It is not a ‘wrapper’ package that simply sources functions from another source, but an integrated set of functions and semantics that does to some extent rely on metafor. The primary strengths include its direct and straightforward implementation with minimal (source) lines of code to do an analysis. Provided one has secured the derived data from the studies and organized into a dataframe with vectors as each key argument within the main meta‐model fitting functions, statistics are straightforward. The type of response variable such as mean, continuous, or rate is matched to a specific function call such as metamean, metacont, or metarate. This is semantically intuitive and encourages good thinking before statistics because it engenders consideration of the data. The effect size calculation is included in this main function and returns the most prevalent effect size measure typically associated with those data, but it can also be specified as an argument. We encourage all synthesis scientists to check whether the default effect size metric is appropriate and to deeply consider this choice. Choosing an effect size metric is one of the most critical decisions in doing a meta‐analysis, and significant literature is devoted to this topic (Mengersen & Gurevitch, 2013; O'Keefe, 2017; Rosenberg, Rothstein, & Gurevitch, 2013; Rücker, Schwarzer, Carpenter, Binder, & Schumacher, 2010). The primary workflow can thus be a single step if the internal calculations provided in this package are aligned with the research on analyses. Exploration of the model is well articulated with funnel, radial, and forest plots. Z‐scores, significance tests, and heterogeneity statistics are printed in the model summary. Publication bias is also provided as a more in‐depth function entitled metabias within this package. There are two standout functions in this package. The first is a function entitled metagen, and it is a backup, multipurpose tool so to speak that fits a generic inverse variance meta‐analysis. This is a handy tool for user‐calculated effect size measures and for the exploration of statistical trends with reduced data or model assumptions. In some fields, there are specific effect size estimates such as the ‘relative intensity of interaction’ effect size metric now common in plant interaction studies (Armas, Ordiales, & Pugnaire, 2004) that this function provides a robust, easy‐to‐fit capacity for statistics. The second standout function is bubble.metareg for a quick, visual exploration of the outcome of a meta‐regression (following the application of the function metareg in this package). It is useful in contemporary data science to use visualization as a means to understand data (Grolemund & Wickham, 2016), but statistical packages do not always provide the means to easily iterate between statistics or model fitting and visualization. In summary, excepting unique data or statistical issues, this package is directly implemented and effective.

Metafor is a more comprehensive package in many respects. This package includes 76 functions, 36 datasets (Appendix S1), a vignette (Viechtbauer, 2010), flowchart as secondary vignette (https://cran.r‐project.org/web/packages/metafor/vignettes/diagram.pdf), and website (http://www.metafor‐project.org/doku.php). The package was last updated on 19 March 2020 (Version 2.4‐0). The text ‘Meta‐analysis with R’ also describes implementation of this package (Schwarzer et al., 2015) but to a lesser extent than the textbook supporting meta. The depth of the package metafor provides greater capacities relative to the meta package but does come at the expense of a steeper initial learning curve. Completing a meta‐analysis using this package requires an additional step, that is, effect sizes must be calculated a priori, not within the model fitting process. This is facilitated with the standalone function escalc, and it can return a wide range of effect sizes measures. Thus, the two‐step process begins with firstly compiling and aggregating the derived dataframe to an effect size table then secondly fitting a model. The data structure is also a bit more rigid for the model fitting, and the nomenclature for this subset of functions is written to parallel more traditional general linear model fitting from conventional statistics. This is both a strength and limitation because one must plan the model to fit in advance and learn the function and arguments, but it is also an advantage as well because model specification uses the familiar notation of tilde. Fitting the best meta‐model is as important at the tool you choose (Jackson, Law, Stijnen, Viechtbauer, & White, 2018; Langan et al., 2019), and in some cases, will determine it because you need a different package. Consequently, similar to the pause for research on the effect size metric selected, this is a natural point in a workflow using metafor to examine your model. Once complete, model fitting is based on the type of model in the call such a random or fixed effects and not on the type of the response data as in meta package. Here, it is more akin to conventional general linear model fitting for those familiar with these functions in R. If the model is more complex with moderators, then this can be directly included in the model fit here via a mods argument whereas in the meta package the model is updated with moderators in a subsequent step. This suggests that if moderators or covariates in the main model are likely relevant to the analyses, then metafor is a stronger starting point. The goal in either instance should not be p‐hacking (Head, Holman, Lanfear, Kahn, & Jennions, 2015) but to fit the most appropriate model to describe the systems summarized in the literature synthesis striking a balance between parsimony and ecological/evolutionary representativeness (Preacher, 2006). The model summary from metafor prints Z‐scores, significance tests, and two sets of heterogeneity estimates. Forest and radial plots are also provided as additional functions. Publication bias statistical estimator functions include trimfill and ranktest. Standout elements of this package include GOSH plots that provide a graphical display of study heterogeneity (Olkin, Dahabreh, & Trikalinos, 2012) and the enhanced model fitting capacities such as the function fitstats that provides log‐likelihood estimates and AIC or BIC scores on meta‐analysis objects. This package requires a deeper focus on model fitting, and while there is additional effort in specifying the data at the onset of the workflow, the rewards in subsequent tools to handle models are significant.

Models are powerful and typically necessary tools to better understand data. Conservatively, a model is a mechanism to explore uncertainty and assign weight to an observed pattern in the data (Glad & Hjort, 2016). Any statistical test, albeit simple, is thus a model to help describe a trend or difference. Less generously, it has been proposed that all models are wrong but that some are useful (Stouffer, 2019). In this vein, it is not unreasonable to examine the outputs of similar packages to ensure that reported findings are relatively consistent such as meta, metafor, or other relevant packages in R (Lortie et al., 2020). This is also an important consideration that can better advance replication science, that is, can we repeat analyses within our fields for the exact same data using different tools and reach similar conclusions (Kelly, 2019). A Cochrane dataset commonly used in teaching and many texts on meta‐analyses (Spooner, Saunders, & Rowe, 2002) was tested in both meta and metafor to ensure that outputs were similar (Appendix S2). This is an ideal teaching dataset because it is tidy, direct, and without covariates. Nonetheless, it is representative for ecologists conceptually because there were a treatment and control with clear reporting of mean and standard errors common in the reporting of the primary studies that we synthesize. The reported statistics from simple univariate, random models were nearly identical from these two R packages. The purpose of this exercise is to illustrate that it is worthwhile to consider adding contrasts to a meta‐analytical workflow. This worked example also provides a concrete instance of the differences in coding and semantics associated with the implementation of each package described above. However, the intent here was not to comprehensively examine sensitivity between these two options nor rigorously examine model similarities. We also do not mean to imply that these packages will always return similar outputs for more advanced models or for different data. If external validity and higher levels of certainty in the strength of findings and robustness of statistics are needed, Stata is an additional resource to triple‐check your work for meta‐analyses (or if you do not work in R, an alternative ecosystem). It is a common application used in many disciplines such as the social sciences and medicine for meta‐analyses and rigorously tested and reviewed by experts. There are extensive resources to support meta‐analyses in Stata including descriptions of workflows and worked examples (Chaimani, Mavridis, & Salanti, 2014). It was also recently updated, December 2019 (Version 16), includes 13 key functions for meta‐analyses, a reference manual specific to meta‐analyses (StataCorp, 2019), and adopts a workflow akin to metafor. Prepare the data and calculate effect sizes, run a meta‐analysis on these effect sizes and inspect summary statistics, explore heterogeneity, and check for small‐study effects and publication bias. Tools are provided for each step. We used the same Cochrane data in Stata 16 and repeated the analysis (Appendix S3, restricted maximum‐likelihood estimate also specified). The reported estimates, *p*‐value, and confidence limits were also nearly identical to both R packages in this instance. Collectively, this suggests that the synthesis scientist has many viable options and that checks between tools are within reach to explore sensitivity.

## 4 CONCLUSIONS

Statistics are sometimes about preferences and thinking styles (Hector, 2017), and scientific synthesis is both an art and a science (Lortie & Bonte, 2016). Trade‐offs are also common in adopting one ecosystem, analysis tool, or specific package for data wrangling and analyses. If more rapid, less specified, general meta‐analyses are the goal—the package meta is a direct means to an end. Moderators are added post hoc, and the first model fit is a single, intuitive process. Meta‐regression is viable and interaction terms can be included. The generic meta‐analysis function is a useful tool for nonstandard effect size metrics. Metafor requires the effect size compilation a priori and is thus a bit more coding to prepare for the meta‐model. However, deeper and more complex model fits are inherent in the semantics of these functions. If the synthesist does have not effect size measure in hand or wishes to calculate effect sizes measures but not for meta‐models, the escalc function is invaluable in this package. In summary, both packages provide the capacity for basic and advanced meta‐analyses but more advanced modeling is likely worth the commitment to metafor. The language R is not the only ecosystem that supports meta‐analyses. There are other applications such as Stata that have the capacity to do meta‐analyses and validate/generate models. Depending on your analytical workflows and the need to validate findings, the learning curve in Stata is relatively shallow. Nonetheless, replication science is best realized through documented, published, and open code, and the usefulness in meta‐analyses is not just in the synthesis of the research but in the process of doing the scientific synthesis. Documenting academic practices is a meaningful step in advancing more transparent practices in reporting the process of all science including synthesis.

## CONFLICT OF INTEREST

None declared.

## AUTHOR CONTRIBUTION

**Christopher J. Lortie:** Conceptualization (lead); Data curation (lead); Formal analysis (lead); Funding acquisition (lead); Investigation (equal); Methodology (equal); Project administration (equal); Validation (equal). **Alex Filazzola:** Validation (equal).

### OPEN RESEARCH BADGES

This article has been awarded Open Materials and Open Data Badges. All materials and data are publicly accessible via the Open Science Framework at https://zenodo.org/account/settings/github/repository/cjlortie/Meta_contrasts#

## Supporting information

Appendix S1

Appendix S2

Appendix S3

## Data Availability

All data are available on https://figshare.com/articles/dataset/A_list_of_meta-analysis_functions_and_objects_in_the_R_packages_meta_and_metafor_with_a_contrast_to_Stata/12280943. Methods contrast data also published on https://figshare.com/articles/dataset/Meta_thinking_data_preventing_exercise-induced_bronchoconstriction/7767020 (Lortie, 2019; Lortie & Filazzola, 2020).
