# Supplementary material for: A contrast of meta and metafor packages for meta‐analyses in R
Source: Ecol Evol. 2020 Sep 14;10(20):10916–21. doi: 10.1002/ece3.6747 (PMC7593135; doi:10.1002/ece3.6747)
Supplement: Supplementary file 1 — Appendix S1 [file ECE3-10-10916-s001.pdf]

Appendix A

A list of objects included in each package when loaded into the R environment.

package	object	role	type
meta	amlodipine	Amlodipine for Work Capacity	data
meta	cisapride	Cisapride in Non-Ulcer Dispepsia	data
meta	Fleiss93	Aspirin after Myocardial Infarction	data
meta	Fleiss93cont	Mental Health Treatment	data
meta	Olkin95	Thrombolytic Therapy after Acute Myocardial Infarction	data
meta	smoking	Smoking example	data
meta	woodyplants	Elevated CO_2 and total biomass of woody plants	data
meta	as.data.frame.meta	Additional functions for objects of class meta	function
meta	baujat	Baujat plot to explore heterogeneity in meta-analysis	function
meta	bubble	Bubble plot to display the result of a meta-regression	function
meta	ci	Calculation of confidence intervals (based on normal...	function
meta	forest	Forest plot to display the result of a meta-analysis	function
meta	funnel	Plot to assess funnel plot asymmetry	function
meta	gs	Get default for a meta-analysis setting.	function
meta	labbe	L'Abbe plot for meta-analysis with binary outcomes	function
meta	metabias	Test for funnel plot asymmetry	function
meta	metabin	Meta-analysis of binary outcome data	function
meta	metabind	Combine meta-analysis objects	function
meta	metacont	Meta-analysis of continuous outcome data	function
meta	metacor	Meta-analysis of correlations	function
meta	metacr	Meta-analysis of outcome data from Cochrane review	function
meta	metacum	Cumulative meta-analysis	function
meta	metagen	Generic inverse variance meta-analysis	function
meta	metainc	Meta-analysis of incidence rates	function

(continued)

package	object	role	type
meta	metainf	Influence analysis in meta-analysis using leave-one-out...	function
meta	metamean	Meta-analysis of single means	function
meta	meta-package	meta: Brief overview of methods and general hints	function
meta	metaprop	Meta-analysis of single proportions	function
meta	metarate	Meta-analysis of single incidence rates	function
meta	metareg	Meta-regression	function
meta	print.meta	Print and summary method for objects of class meta	function
meta	print.rm5	Print and summary methods for objects of class rm5	function
meta	read.mtv	Import RevMan 4 data files (.mtv)	function
meta	read.rm5	Import RevMan 5 data files (.csv)	function
meta	settings.meta	Print and change default settings to conduct and print or...	function
meta	trimfill	Trim-and-fill method to adjust for bias in meta-analysis	function
meta	update.meta	Update a meta-analysis object	function
meta	weights.meta	Calculate absolute and percentage weights for meta-analysis	function
meta	nnt	Calculate the number needed to treat from estimated risk difference	function
meta	or2smd	Conversion from log odds ratio to standardised mean difference	function
metafor	dat.bangertdrowns2004	Studies on the Effectiveness of Writing-to-Learn...	data
metafor	dat.begg1989	Studies on Bone-Marrow Transplantation versus Chemotherapy...	data
metafor	dat.berkey1998	Studies on Treatments for Periodontal Disease	data
metafor	dat.bonett2010	Studies on the Reliability of the CES-D Scale	data
metafor	dat.bourassa1996	Studies on the Association between Handedness and...	data
metafor	dat.colditz1994	Studies on the Effectiveness of the BCG Vaccine Against...	data
metafor	dat.collins1985a	Studies on the Treatment of Upper Gastrointestinal Bleeding...	data

(continued)

package	object	role	type
metafor	dat.collins1985b	Studies on the Effects of Diuretics in Pregnancy	data
metafor	dat.curtis1998	Studies on the Effects of Elevated CO2 Levels on Woody Plant...	data
metafor	dat.debruin2009	Studies on Standard Care Quality and HAART-Adherence	data
metafor	dat.egger2001	Studies on the Effectiveness of Intravenous Magnesium in...	data
metafor	dat.fine1993	Studies on Radiation Therapy with or without Adjuvant...	data
metafor	dat.gibson2002	Studies on the Effectiveness of Self-Management Education and...	data
metafor	dat.hackshaw1998	Studies on Lung Cancer Risk from ETS Exposure	data
metafor	dat.hart1999	Studies on the Effectiveness of Warfarin for Preventing...	data
metafor	dat.hasselblad1998	Studies on the Effectiveness of Counseling for Smoking...	data
metafor	dat.hine1989	Studies on Prophylactic Use of Lidocaine After a Heart Attack	data
metafor	dat.ishak2007	Studies on Deep-Brain Stimulation	data
metafor	dat.konstantopoulos2011	Studies on the Effects of Modified School Calendars on...	data
metafor	dat.laopaiboon2015	Studies on the Effectiveness of Azithromycin for Treating...	data
metafor	dat.lee2004	Studies on Acupoint P6 Stimulation for Preventing Nausea	data
metafor	dat.li2007	Studies on the Effectiveness of Intravenous Magnesium in...	data
metafor	dat.linde2005	Studies on the Effectiveness of St. John's Wort for Treating...	data
metafor	dat.mcdaniel1994	Studies on the Validity of Employment Interviews	data
metafor	dat.molloy2014	Studies on the Relationship between Conscientiousness and...	data
metafor	dat.nielweise2007	Studies on Anti-Infective-Treated Central Venous Catheters...	data
metafor	dat.nielweise2008	Studies on Anti-Infective-Treated Central Venous Catheters...	data
metafor	dat.normand1999	Studies on the Length of Hospital Stay of Stroke Patients	data
metafor	dat.pagliaro1992	Studies on the Effectiveness of Nonsurgical Treatments in...	data

(continued)

package	object	role	type
metafor	dat.pignon2000	Studies on the Effectiveness of Locoregional Treatment plus...	data
metafor	dat.pritz1997	Studies on the Effectiveness of Hyperdynamic Therapy for...	data
metafor	dat.raudenbush1985	Studies on Assessing the Effects of Teacher Expectations on...	data
metafor	dat.riley2003	Studies on MYC-N as a Prognostic Marker for Neuroblastoma	data
metafor	dat.senn2013	Studies on the Effectiveness of Glucose-Lowering Agents	data
metafor	dat.yusuf1985	Studies of Beta Blockers During and After Myocardial...	data
metafor	addpoly	Add Polygons to Forest Plots	function
metafor	addpoly.default	Add Polygons to Forest Plots (Default Method)	function
metafor	addpoly.rma	Add Polygons to Forest Plots (Method for 'rma' Objects)	function
metafor	anova.rma	Likelihood Ratio and Wald-Type Tests for 'rma' Objects	function
metafor	baujat	Baujat Plots for 'rma' Objects	function
metafor	bldiag	Construct Block Diagonal Matrix	function
metafor	blup	Best Linear Unbiased Predictions for 'rma.uni' Objects	function
metafor	coef.permutest.rma.uni	Extract the Model Coefficient Table from 'permutest.rma.uni'...	function
metafor	coef.rma	Extract the Model Coefficients and Coefficient Table from...	function
metafor	confint.rma	Confidence Intervals for 'rma' Objects	function
metafor	cumul	Cumulative Meta-Analysis for 'rma' Objects	function
metafor	escalc	Calculate Effect Sizes and Outcome Measures	function
metafor	fitstats	Fit Statistics and Information Criteria for 'rma' Objects	function
metafor	fitted.rma	Fitted Values for 'rma' Objects	function
metafor	forest	Forest Plots	function
metafor	forest.cumul.rma	Forest Plots (Method for 'cumul.rma' Objects)	function
metafor	forest.default	Forest Plots (Default Method)	function
metafor	forest.rma	Forest Plots (Method for 'rma' Objects)	function
metafor	fsn	Fail-Safe N Analysis (File Drawer Analysis)	function
metafor	funnel	Funnel Plots	function
metafor	gosh	GOSH Plots for 'rma' Objects	function

(continued)

package	object	role	type
metafor	hc	Meta-Analysis based on the Method by Henmi and Copas (2010)	function
metafor	influence.rma.mv	Outlier and Influential Case Diagnostics for 'rma.mv' Objects	function
metafor	influence.rma.uni	Outlier and Influential Case Diagnostics for 'rma.uni'...	function
metafor	labbe	L'Abbe Plots for 'rma' Objects	function
metafor	leave1out	Leave-One-Out Diagnostics for 'rma' Objects	function
metafor	llplot	Likelihood Plot of a Parameter Corresponding to an Effect...	function
metafor	metafor.news	Read News File of the Metafor Package	function
metafor	metafor-package	metafor: A Meta-Analysis Package for R	function
metafor	methods.escalc	Methods for 'escalc' Objects	function
metafor	methods.list.rma	Methods for 'list.rma' Objects	function
metafor	model.matrix.rma	Model Matrix for 'rma' Objects	function
metafor	permutest	Permutation Tests for 'rma.uni' Objects	function
metafor	plot.cumul.rma	Plot Method for 'cumul.rma' Objects	function
metafor	plot.gosh.rma	Plot Method for 'gosh.rma' Objects	function
metafor	plot.infl.rma.uni	Plot Method for 'infl.rma.uni' Objects	function
metafor	plot.rma	Plot Method for 'rma' Objects	function
metafor	predict.rma	Predicted Values for 'rma' Objects	function
metafor	print.anova.rma	Print Method for 'anova.rma' Objects	function
metafor	print.confint.rma	Print Methods for 'confint.rma' and 'list.confint.rma'...	function
metafor	print.escalc	Print and Summary Methods for 'escalc' Objects	function
metafor	print.fsn	Print Method for 'fsn' Objects	function
metafor	print.gosh.rma	Print Method for 'gosh.rma' Objects	function
metafor	print.hc.rma.uni	Print Method for 'hc.rma.uni' Objects	function
metafor	print.list.rma	Print method for 'list.rma' Objects	function
metafor	print.permutest.rma.uni	Print Method for 'permutest.rma.uni' Objects	function
metafor	print.ranktest.rma	Print Method for 'ranktest.rma' Objects	function
metafor	print.regtest.rma	Print Method for 'regtest.rma' Objects	function

(continued)

package	object	role	type
metafor	print.rma	Print and Summary Methods for 'rma' Objects	function
metafor	print.robust.rma	Print Method for 'robust.rma' Objects	function
metafor	profile.rma.uni	Profile Plots for 'rma' Objects	function
metafor	qqnorm.rma.uni	Normal QQ Plots for 'rma' Objects	function
metafor	radial	Radial (Galbraith) Plots for 'rma' Objects	function
metafor	ranef	Best Linear Unbiased Predictions for 'rma.uni' and 'rma.mv'...	function
metafor	ranktest	Rank Correlation Test for Funnel Plot Asymmetry	function
metafor	regtest	Regression Test for Funnel Plot Asymmetry	function
metafor	replmiss	Replace Missing Values in a Vector	function
metafor	reporter	Dynamically Generated Analysis Reports for 'rma.uni' Objects	function
metafor	residuals.rma	Residual Values based on 'rma' Objects	function
metafor	rma.glmm	Meta-Analysis via Generalized Linear (Mixed-Effects) Models	function
metafor	rma.mh	Meta-Analysis via the Mantel-Haenszel Method	function
metafor	rma.mv	Meta-Analysis via Multivariate/Multilevel Linear...	function
metafor	rma.peto	Meta-Analysis via Peto's Method	function
metafor	rma.uni	Meta-Analysis via Linear (Mixed-Effects) Models	function
metafor	robust	(Cluster) Robust Tests and Confidence Intervals for 'rma'...	function
metafor	simulate.rma	Simulate Method for 'rma' Objects	function
metafor	to.long	Convert Data from Vector to Long Format	function
metafor	to.table	Convert Data from Vector to Table Format	function
metafor	transf	Transformation Function	function
metafor	trimfill	Trim and Fill Analysis for 'rma.uni' Objects	function
metafor	update.rma	Model Updating for 'rma' Objects	function
metafor	vcov.rma	Extract Various Types of Variance-Covariance Matrices from...	function
metafor	vif	Variance Inflation Factors for 'rma' Objects	function

(continued)

package	object	role	type
metafor	weights.rma.uni	Compute Weights for 'rma' Objects	function
metafor	to.wide	Converts data given in long format to a wide format.	function
metafor	contrmat	Constructs a matrix that contrasts two groups	function
metafor	dat.graves2010	Sudies on the effectiveness of injected vaccines against cholera	data
