# Supplementary material for: A contrast of meta and metafor packages for meta‐analyses in R
Source: Ecol Evol. 2020 Sep 14;10(20):10916–21. doi: 10.1002/ece3.6747 (PMC7593135; doi:10.1002/ece3.6747)
Supplement: Supplementary file 2 — Appendix S2 [file ECE3-10-10916-s002.pdf]

Appendix B

A contrast of analyses done using the R packages meta and metafor.

Contrasted meta and metafor using the classic Cochrane bronchoconstriction dataset used in many texts to demonstrate meta-analsysis. Here is a link to these data as a tidy csv files on figshare.

Meta

```
#load primary libraries
library(tidyverse) #tidy functions to read and format data
library(meta) #meta package current version effective March 2020

#Classic bronchoconstriction data from Cochrane
data <- read_csv(url("https://ndownloader.figshare.com/files/14460386"))

#continuous data
m1 <- metacont(Ne, Me, Se, Nc, Mc, Sc, data = data, sm = "SMD")
summary(m1)
```

```
## Number of studies combined: k = 17
##
##                          SMD             95%-CI      z  p-value
## Fixed effect model   -1.0583 [-1.2537; -0.8630] -10.62 < 0.0001
## Random effects model -1.0694 [-1.2813; -0.8575]  -9.89 < 0.0001
##
## Quantifying heterogeneity:
##  tau^2 = 0.0265 [0.0000; 0.3559]; tau = 0.1629 [0.0000; 0.5966];
##  I^2 = 13.5% [0.0%; 50.2%]; H = 1.08 [1.00; 1.42]
##
## Test of heterogeneity:
##      Q d.f. p-value
##  18.49   16  0.2959
##
## Details on meta-analytical method:
## - Inverse variance method
## - DerSimonian-Laird estimator for tau^2
## - Jackson method for confidence interval of tau^2 and tau
## - Hedges' g (bias corrected standardised mean difference)
```

```
#viz
forest(m1, layout = "JAMA")
```

Source	SMD (95% CI)
1	−1.04 [−2.04; −0.04]
2	−2.48 [−3.88; −1.09]
3	−1.14 [−2.01; −0.26]
4	−1.37 [−2.27; −0.46]
5	−1.02 [−1.88; −0.16]
6	−0.39 [−1.16; 0.39]
7	−1.93 [−2.92; −0.93]
8	−0.46 [−1.09; 0.17]
9	−1.38 [−2.16; −0.60]
10	−1.18 [−2.06; −0.30]
11	−1.88 [−2.83; −0.93]
12	−0.87 [−1.58; −0.17]
13	−0.80 [−1.83; 0.23]
14	−1.12 [−1.73; −0.51]
15	−1.08 [−1.76; −0.39]
16	−0.75 [−1.55; 0.05]
17	−1.01 [−1.67; −0.35]
Total (fixed effect)	−1.06 [−1.25; −0.86]
Total (random effects)	−1.07 [−1.28; −0.86]

Heterogeneity: χ^2_{16} = 18.49 (P = .30), I^2 = 13%

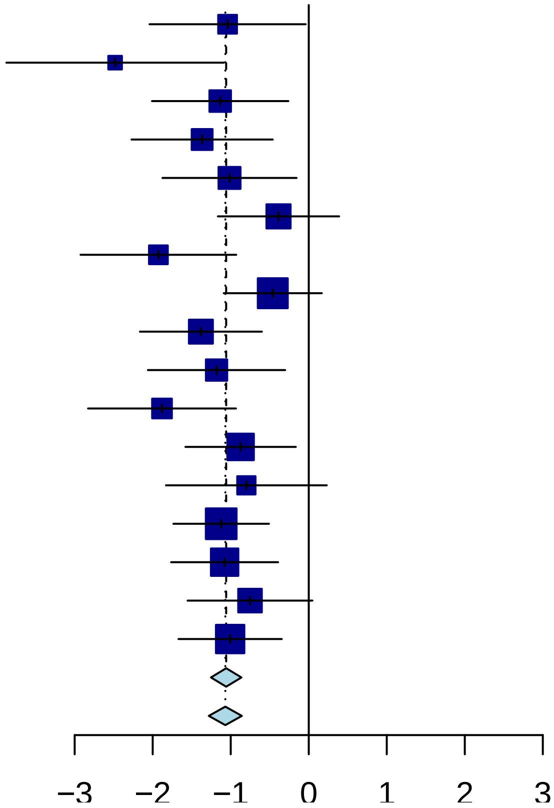


```
metabias(m1)
```

```
##
##  Linear regression test of funnel plot asymmetry
##
## data:  m1
## t = -2.8469, df = 15, p-value = 0.01224
## alternative hypothesis: asymmetry in funnel plot
## sample estimates:
##       bias    se.bias      slope
## -3.2230339  1.1321337  0.2416821
```

```
funnel(m1)
```

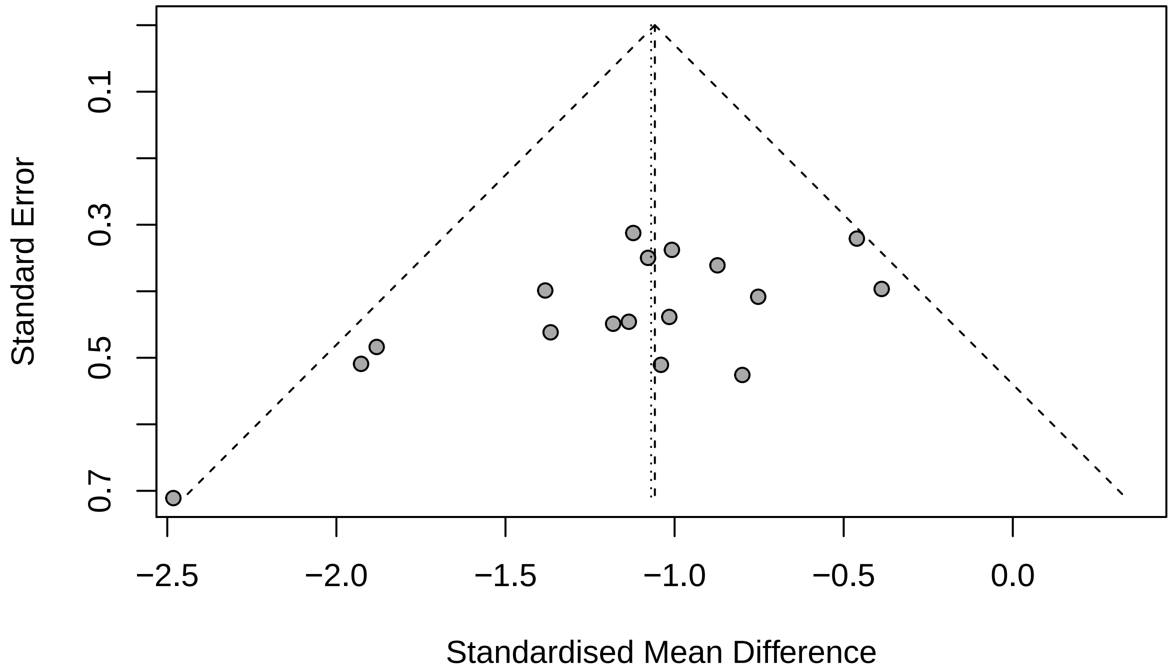

Standard Error
0.1
0.3
0.5
0.7
−2.5
−2.0
−1.5
−1.0
−0.5
0.0
Standardised Mean Difference


```
radial(m1)
```

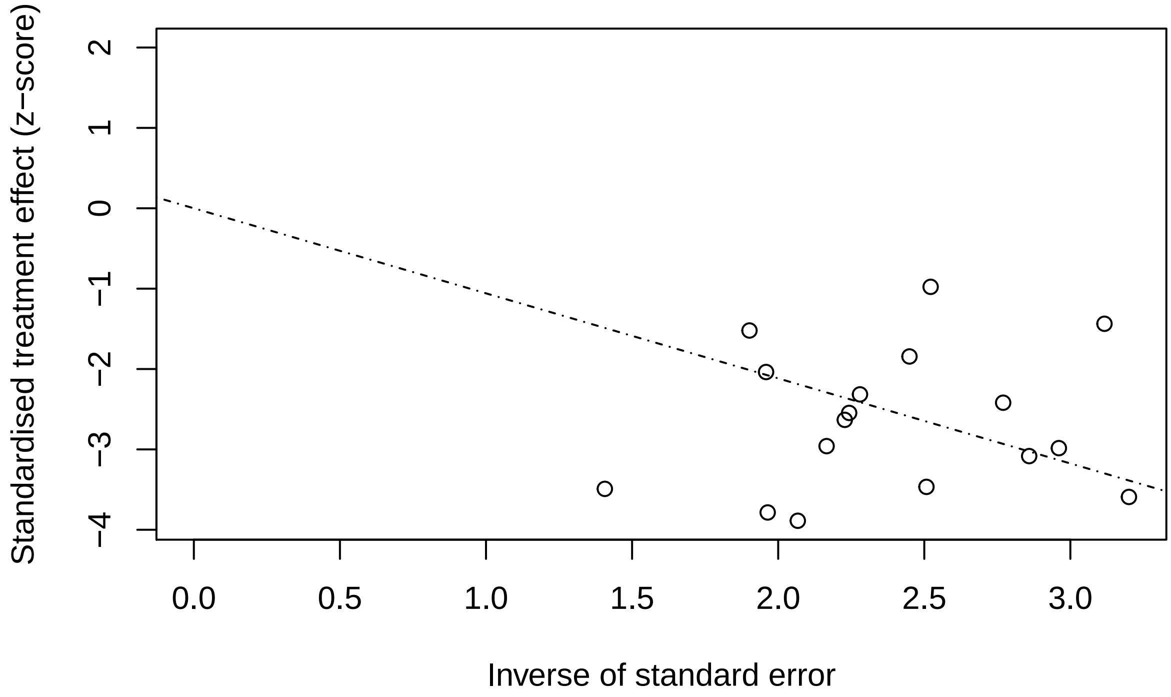

Standardised treatment effect (z−score)
2
1
0
−1
−2
−3
−4
0.0
0.5
1.0
1.5
2.0
2.5
3.0
Inverse of standard error

Metafor

```
#load primary libraries
library(tidyverse) #tidy functions to read and format data
library(metafor) #metafor package current version effective March 2020

#Classic bronchoconstriction data from Cochrane
data <- read_csv(url("https://ndownloader.figshare.com/files/14460386"))

#calculate effect sizes
data <- escalc(measure = "SMD", m1i=Me,m2i=Mc,sd1i=Se,sd2i=Sc,n1i=Ne,n2i=Nc, data=data,var.names=c("SMD

#fit model
#m2 <- rma(yi=SMD, vi=SMD_var, mods = ~study.ID, data = data)
m2 <- rma.uni(yi=SMD, vi=SMD_var, data = data, method = "REML") #restricted maximum-likelihood estimate
summary(m2)
```

```
##
## Random-Effects Model (k = 17; tau^2 estimator: REML)
##
##   logLik  deviance       AIC       BIC      AICc
## -10.5948   21.1896   25.1896   26.7348   26.1127
##
## tau^2 (estimated amount of total heterogeneity): 0.0142 (SE = 0.0613)
## tau (square root of estimated tau^2 value):      0.1193
## I^2 (total heterogeneity / total variability):   7.88%
## H^2 (total variability / sampling variability):  1.09
##
## Test for Heterogeneity:
## Q(df = 16) = 19.4639, p-val = 0.2453
##
## Model Results:
##
## estimate      se      zval    pval    ci.lb    ci.ub
##  -1.0727  0.1032  -10.3919  <.0001  -1.2750  -0.8703  ***
##
## ---
## Signif. codes:  0 '***' 0.001 '**' 0.01 '*' 0.05 '.' 0.1 ' ' 1
```

```
#Viz
plot(m2)
```

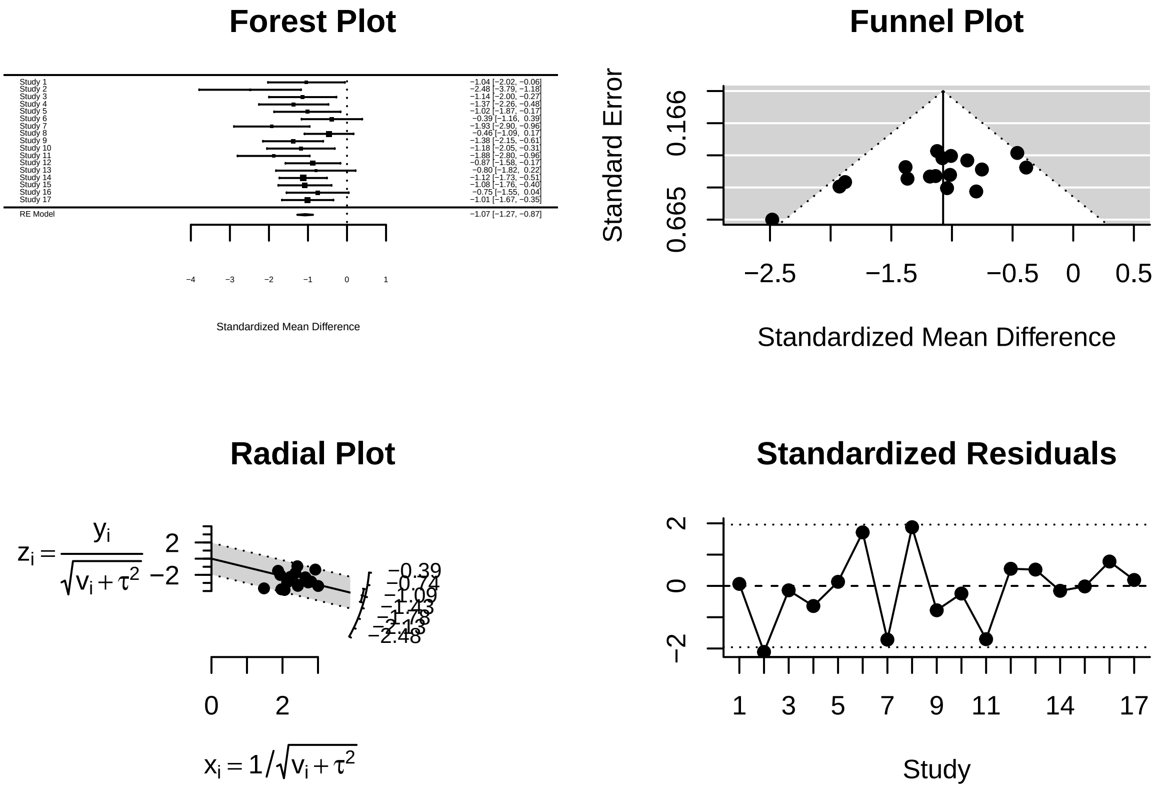
Forest Plot
Study 1 −1.04 [−2.02, −0.06]
Study 2 −2.48 [−3.79, −1.18]
Study 3 −1.14 [−2.00, −0.27]
Study 4 −1.37 [−2.26, −0.48]
Study 5 −1.02 [−1.87, −0.17]
Study 6 −0.39 [−1.16, 0.39]
Study 7 −1.93 [−2.90, −0.96]
Study 8 −0.46 [−1.09, 0.17]
Study 9 −1.38 [−2.15, −0.61]
Study 10 −1.18 [−2.05, −0.31]
Study 11 −1.88 [−2.80, −0.96]
Study 12 −0.87 [−1.58, −0.17]
Study 13 −0.80 [−1.82, 0.22]
Study 14 −1.12 [−1.73, −0.51]
Study 15 −1.08 [−1.76, −0.40]
Study 16 −0.75 [−1.55, 0.04]
Study 17 −1.01 [−1.67, −0.35]
RE Model −1.07 [−1.27, −0.87]
−4 −3 −2 −1 0 1
Standardized Mean Difference
Funnel Plot
Standard Error
0.166
0.665
−2.5 −1.5 −0.5 0 0.5
Standardized Mean Difference
Radial Plot
$z_i = \frac{y_i}{\sqrt{v_i + \tau^2}}$
2
−2
−0.39
−0.74
−1.09
−2.48
0 2
$x_i = 1/\sqrt{v_i + \tau^2}$
Standardized Residuals
2
0
−2
1 3 5 7 9 11 14 17
Study

Compiled contrasts

```
#compile random model outputs for contrast with metafor
estimate <- as_data_frame(m1$TE.random)
se <- as_data_frame(m1$seTE.random)
zval <- as_data_frame(m1$zval.random)
pval <- as_data_frame(m1$pval.random)
ci.lb <- as_data_frame(m1$lower.random)
ci.ub <- as_data_frame(m1$upper.random)
m1_tidy <- bind_cols(estimate, se, zval, pval, ci.lb, ci.ub) %>%
  rename(estimate = value, se = value1, zval = value2, pval = value3, ci.lb = value4, ci.ub = value5)

#compile output into table for contrast with meta
m2_tidy <- coef(summary(m2))
#m2_tidy

#compile key outputs
contrast_tidy <- bind_rows(m1_tidy, m2_tidy) %>%
  mutate(source = c("meta", "metafor")) %>%
  select(source, everything())

knitr::kable(contrast_tidy)
```

source	estimate	se	zval	pval	ci.lb	ci.ub
meta	-1.069383	0.1081185	-9.890843	0	-1.281291	-0.8574745
metafor	-1.072657	0.1032201	-10.391944	0	-1.274965	-0.8703497
