# Supplementary material for: A contrast of meta and metafor packages for meta‐analyses in R
Source: Ecol Evol. 2020 Sep 14;10(20):10916–21. doi: 10.1002/ece3.6747 (PMC7593135; doi:10.1002/ece3.6747)
Supplement: Supplementary file 3 — Appendix S3 [file ECE3-10-10916-s003.pdf]

# Appendix C

**An example of a meta-analysis conducted in Stata 16.**

The meta-analysis was conducted using the classic Cochrane bronchoconstriction dataset used in many texts to demonstrate meta-analysis. Below are the results from the meta-analysis including bias tests and funnel plots.

. meta esize ne me se nc mc sc, esize(hedgesg)

| source | estimate | se | zval | pval | ci.lb | ci.ub |
|---|---|---|---|---|---|---|
| Stata | -1.08 | 0.10 | -10.39 | 0 | -1.28 | -0.87 |

. meta summarize

```
  Effect-size label:  Hedges's g
        Effect size:  _meta_es
          Std. Err.:  _meta_se

Meta-analysis summary                     Number of studies =     17
Random-effects model                      Heterogeneity:
Method: REML                                          tau2 =  0.0228
                                                    I2 (%) =   12.60
                                                        H2 =    1.14
```

| Study | Hedges's g | [95% Conf. | Interval] | % Weight |
|---|---|---|---|---|
| Study 1 | -1.040 | -1.984 | -0.097 | 4.22 |
| Study 2 | -2.482 | -3.746 | -1.218 | 2.44 |
| Study 3 | -1.135 | -1.972 | -0.299 | 5.23 |
| Study 4 | -1.367 | -2.230 | -0.503 | 4.94 |
| Study 5 | -1.016 | -1.840 | -0.191 | 5.37 |
| Study 6 | -0.388 | -1.140 | 0.364 | 6.31 |
| Study 7 | -1.927 | -2.873 | -0.982 | 4.20 |
| Study 8 | -0.461 | -1.077 | 0.155 | 8.83 |
| Study 9 | -1.382 | -2.138 | -0.627 | 6.26 |
| Study 10 | -1.182 | -2.023 | -0.340 | 5.18 |
| Study 11 | -1.881 | -2.784 | -0.978 | 4.56 |
| Study 12 | -0.873 | -1.562 | -0.185 | 7.34 |
| Study 13 | -0.800 | -1.767 | 0.167 | 4.03 |
| Study 14 | -1.122 | -1.722 | -0.522 | 9.20 |
| Study 15 | -1.079 | -1.747 | -0.410 | 7.72 |
| Study 16 | -0.753 | -1.525 | 0.019 | 6.03 |
| Study 17 | -1.008 | -1.654 | -0.362 | 8.15 |
| theta | -1.076 | -1.279 | -0.873 | |

```
Test of theta = 0: z = -10.39                      Prob > |z| = 0.0000
Test of homogeneity: Q = chi2(16) = 20.51            Prob > Q = 0.1981
```

. meta forestplot

| Study | Treatment N | Treatment Mean | Treatment SD | Control N | Control Mean | Control SD | Hedges's g with 95% CI | Weight (%) |
|---|---|---|---|---|---|---|---|---|
| Study 1 | 9 | 18.9 | 17.7 | 9 | 38.9 | 18.9 | -1.04 [ -1.98, -0.10] | 4.22 |
| Study 2 | 8 | 10.27 | 7.02 | 8 | 34.43 | 10.96 | -2.48 [ -3.75, -1.22] | 2.44 |
| Study 3 | 12 | 29.83 | 15.95 | 12 | 48.08 | 15.08 | -1.14 [ -1.97, -0.30] | 5.23 |
| Study 4 | 12 | 21.3 | 13.1 | 12 | 39.7 | 12.9 | -1.37 [ -2.23, -0.50] | 4.94 |
| Study 5 | 12 | 12 | 14.6 | 12 | 26.2 | 12.3 | -1.02 [ -1.84, -0.19] | 5.37 |
| Study 6 | 13 | 13.54 | 13.85 | 13 | 20.77 | 21.46 | -0.39 [ -1.14, 0.36] | 6.31 |
| Study 7 | 12 | 17.5 | 13.1 | 12 | 47.2 | 16.47 | -1.93 [ -2.87, -0.98] | 4.20 |
| Study 8 | 20 | 15.7 | 13.1 | 20 | 22.7 | 16.47 | -0.46 [ -1.08, 0.15] | 8.83 |
| Study 9 | 16 | 15.83 | 13.43 | 16 | 38.36 | 18.01 | -1.38 [ -2.14, -0.63] | 6.26 |
| Study 10 | 12 | 14.5 | 12.2 | 12 | 31.3 | 15.1 | -1.18 [ -2.02, -0.34] | 5.18 |
| Study 11 | 13 | 10.1 | 8.9 | 13 | 23.5 | 4 | -1.88 [ -2.78, -0.98] | 4.56 |
| Study 12 | 17 | 14.4 | 11.1 | 17 | 27.4 | 17.3 | -0.87 [ -1.56, -0.18] | 7.34 |
| Study 13 | 8 | 14.8 | 18.6 | 8 | 31.4 | 20.6 | -0.80 [ -1.77, 0.17] | 4.03 |
| Study 14 | 24 | 15.42 | 8.35 | 24 | 28.46 | 13.84 | -1.12 [ -1.72, -0.52] | 9.20 |
| Study 15 | 19 | 11 | 12.4 | 19 | 26.1 | 14.9 | -1.08 [ -1.75, -0.41] | 7.72 |
| Study 16 | 13 | 15.7 | 16.8 | 13 | 29.6 | 18.9 | -0.75 [ -1.52, 0.02] | 6.03 |
| Study 17 | 20 | 14.1 | 9.5 | 20 | 28.9 | 18 | -1.01 [ -1.65, -0.36] | 8.15 |
| **Overall** | | | | | | | -1.08 [ -1.28, -0.87] | |

Heterogeneity: $\tau^2 = 0.02$, $I^2 = 12.60\%$, $H^2 = 1.14$

Test of $\theta_i = \theta_j$: Q(16) = 20.51, p = 0.20

Test of $\theta = 0$: z = -10.39, p = 0.00

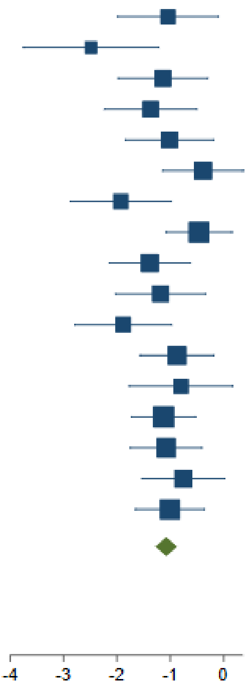


Random-effects REML model

. meta funnelplot

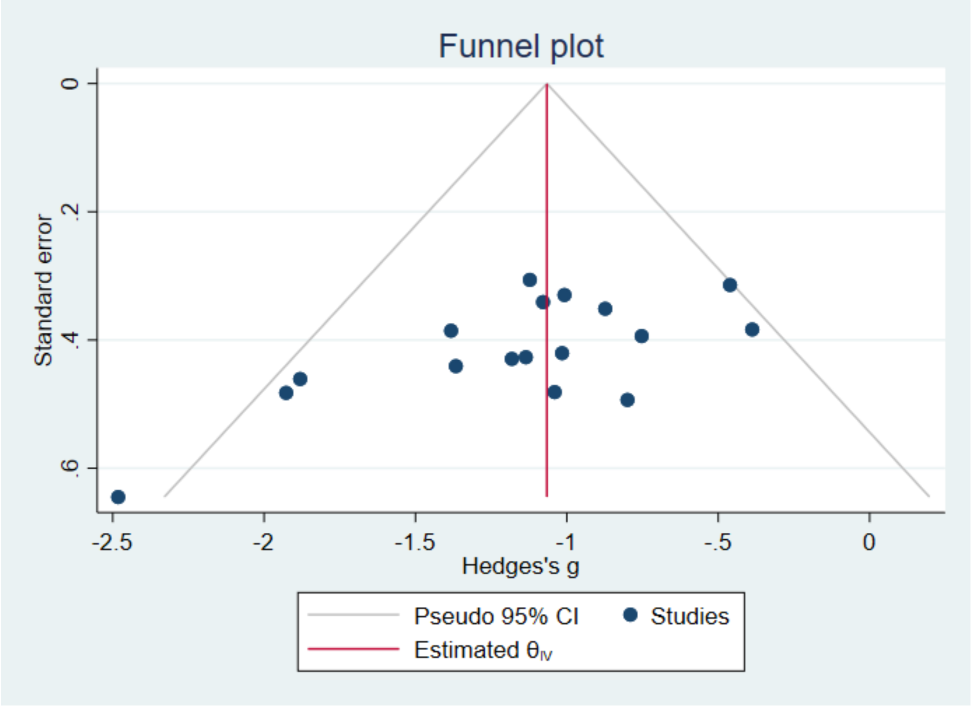

```
. meta bias, egger

  Effect-size label:  Hedges's g
        Effect size:  _meta_es
          Std. Err.:  _meta_se

Regression-based Egger test for small-study effects
Random-effects model
Method: REML

H0: beta1 = 0; no small-study effects
            beta1 =     -3.73
      SE of beta1 =     1.373
                z =     -2.72
       Prob > |z| =    0.0066
```
